# Dose volume histogram analysis of normal structures associated with accelerated partial breast irradiation delivered by high dose rate brachytherapy and comparison with whole breast external beam radiotherapy fields

**DOI:** 10.1186/1748-717X-3-39

**Published:** 2008-11-19

**Authors:** Alexandra J Stewart, Desmond A O'Farrell, Robert A Cormack, Jorgen L Hansen, Atif J Khan, Subhakar Mutyala, Phillip M Devlin

**Affiliations:** 1 St Luke's Cancer Centre, Royal Surrey County Hospital, Guildford, Surrey, UK; 2 Division of Brachytherapy, Department of Radiation Oncology, Dana Faber/Brigham and Women's Cancer Center, Harvard Medical School, Boston, MA, USA; 3 Department of Radiation Oncology, Cancer Institute of New Jersey, New Jersey, USA; 4 Department of Radiation Oncology, Montefiore Medical Center and Albert Einstein College of Medicine in Bronx, New York, USA

## Abstract

**Purpose:**

To assess the radiation dose delivered to the heart and ipsilateral lung during accelerated partial breast brachytherapy using a MammoSite™ applicator and compare to those produced by whole breast external beam radiotherapy (WBRT).

**Materials and methods:**

Dosimetric analysis was conducted on patients receiving MammoSite breast brachytherapy following conservative surgery for invasive ductal carcinoma. Cardiac dose was evaluated for patients with left breast tumors with a CT scan encompassing the entire heart. Lung dose was evaluated for patients in whom the entire lung was scanned. The prescription dose of 3400 cGy was 1 cm from the balloon surface. MammoSite dosimetry was compared to simulated WBRT fields with and without radiobiological correction for the effects of dose and fractionation. Dose parameters such as the volume of the structure receiving 10 Gy or more (V10) and the dose received by 20 cc of the structure (D20), were calculated as well as the maximum and mean doses received.

**Results:**

Fifteen patients were studied, five had complete lung data and six had left-sided tumors with complete cardiac data. Ipsilateral lung volumes ranged from 925–1380 cc. Cardiac volumes ranged from 337–551 cc. MammoSite resulted in a significantly lower percentage lung V30 and lung and cardiac V20 than the WBRT fields, with and without radiobiological correction.

**Conclusion:**

This study gives low values for incidental radiation received by the heart and ipsilateral lung using the MammoSite applicator. The volume of heart and lung irradiated to clinically significant levels was significantly lower with the MammoSite applicator than using simulated WBRT fields of the same CT data sets.

**Trial registration:**

Dana Farber Trial Registry number 03-179

## Background

Accelerated partial breast irradiation (APBI) is increasingly being used as an alternative to whole breast irradiation following wide local excision in selected patients with early stage low-risk breast cancer [1]. The technique is appealing to both physicians and patients due to the decrease in overall treatment time and the reduction in treatment volume. The majority of published series of patients treated with APBI have used brachytherapy [1-17]. Initial data using multiple interstitial catheters using either high dose rate (HDR) or low dose rate (LDR) brachytherapy has shown promising results [12,15,17]. However, interstitial implants can be complex and operator-dependant therefore the MammoSite applicator (Hologic, Bedford, Massachusetts, USA) was developed to make APBI with brachytherapy more accessible and less invasive. Since this is a new technology, there is a paucity of long-term follow-up using this technique. The prospective series with the longest follow-up to date using the MammoSite catheter show low levels of ipsilateral breast recurrence with minimal incidence of tumor bed recurrence [2,14,16].

Direct dosimetric comparisons have been made between different forms of APBI using intensity modulated radiotherapy (IMRT), 3-dimensional conformal external beam radiotherapy (3DCRT) and MammoSite brachytherapy [18]. Dose comparisons have also been made between patients undergoing whole breast external beam radiotherapy (EBRT) and ABPI, simulating the position of a MammoSite catheter within the breast on EBRT CT treatment planning scans [19]. However, data has not been published on direct comparisons of the normal tissue dosimetry for whole breast EBRT and APBI in patients who have a MammoSite applicator in situ. This study examines the dosimetry of the heart and ipsilateral lung in patients undergoing APBI with a MammoSite catheter. The organs at risk (OAR) dosimetry when using the MammoSite catheter was compared with that of reconstructed EBRT fields, taking into consideration the radiobiological characteristics of the MammoSite catheter and the effect of an increased dose per fraction in the APBI treatment regime.

## Methods

### Patient eligibility

Fifteen patients were prospectively enrolled in an institutional review board approved feasibility study. All patients underwent breast-conserving surgery with partial mastectomy and negative sentinel lymph node biopsy or axillary dissection for T1/T2 invasive ductal carcinoma between September 2003 and February 2005. The MammoSite applicator was sited in the tumor cavity either under direct vision intra-operatively or using ultrasound guidance post-operatively.

### Treatment planning

**Figure 1:**
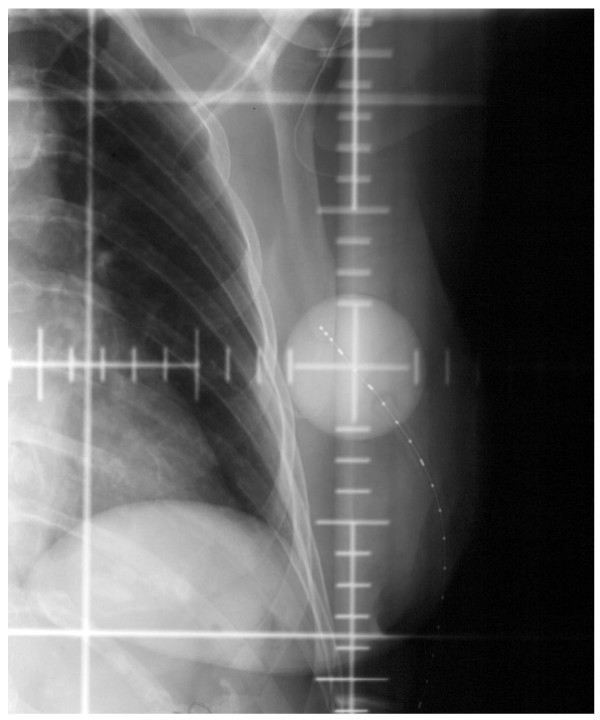
**AP radiograph demonstrating the image reviewed for daily quality assurance measurements of the MammoSite balloon diameter.** The catheter contains a radio-opaque strand with markers at intervals of 1 cm.

**Figure 2:**
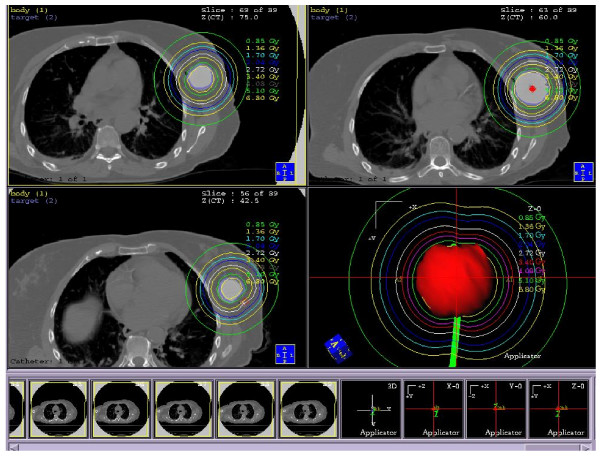
Axial CT images demonstrating the isodose pattern of the MammoSite balloon and surrounding critical normal tissues.

All patients underwent a CT treatment-planning scan following MammoSite balloon insertion. In addition the patients received daily conventional simulation films using fluoroscopy to ensure consistency in balloon diameter, see figure 1. The CT images were transferred to Plato brachytherapy planning system (version 14.2.6, Nucletron BV, Veenendaal, The Netherlands). A dose of 3.4 Gy per fraction for a 10 fraction treatment course was prescribed at 1 cm from the balloon surface. The dose was optimized to 6 points at +/- x, y, z axis positions. Seven to nine dwell positions with 5 mm spacing were used to improve dose homogeneity and decrease the effect of source anisotropy [20], see figure 2.

The source strength, dwell positions and times were transferred with the CT images to the Eclipse treatment planning system (Brachytherapy planning 6.5, Varian Medical Systems, Palo Alto, California, USA) for OAR contouring and dose volume histogram (DVH) analysis. The lungs were contoured from apex to diaphragm, to represent the whole ipsilateral lung within the parietal pleura. The whole heart was contoured, to represent the myocardium. In the absence of intravenous contrast administration, it was not possible to define the left ventricle or coronary arteries. The balloon surface was contoured manually. All contouring was performed by the same practitioner (AJS).

**Figure 3:**
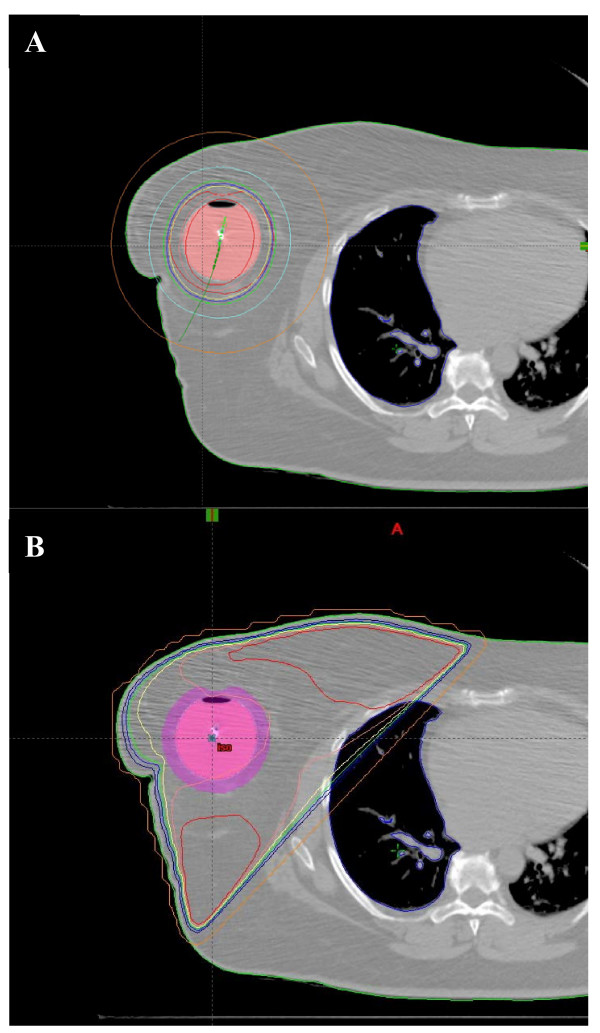
Axial CT images to demonstrate the lung dosimetry at the same level on the same patient showing the MammoSite dosimetry (3A) and the simulated EBRT field dosimetry (3B).

**Figure 4:**
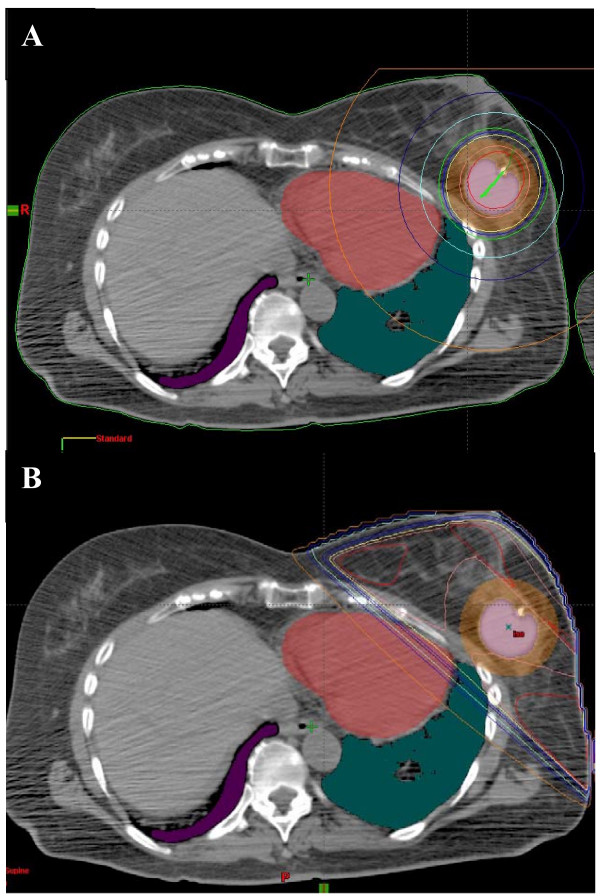
**Axial CT images to demonstrate cardiac dosimetry at the same level on the same patient showing the MammoSite dosimetry (4A) and the simulated EBRT field dosimetry (4B).** The heart is shown in the red colorwash and the lungs in the green colorwash.

Using the same CT treatment planning data, with the inflated MammoSite catheter in situ, a course of fractionated whole breast EBRT was planned using the Pinnacle system (External Beam Planning 6.5 build 7.3.10, Varian Medical Systems, Palo Alto, California, USA). Tangent fields were set up for each patient using standard medial (patient midline) and lateral (mid-axillary line) borders with appropriate collimator angulations to minimize ipsilateral lung volume irradiation. Appropriate anterior and inferior flash was used. Typical tangential weightings and wedge compensators were employed to achieve reasonable homogeneity of dose across the breast tissue. A dose of 50 Gy in 25 fractions over 5 weeks was modeled. DVHs were prepared for the tissues under study. Each patient served as their own internal control with respect to anatomy and therefore EBRT dosimetry and MammoSite dosimetry was compared using identical CT data sets. Figures 3 and 4 demonstrate the MammoSite and EBRT treatment fields and their relationship to lung dosimetry (figures 3A and 3B) and cardiac dosimetry (4A and 4B). WBRT was chosen as a comparator rather than other partial breast irradiation techniques because the only alternative treatment to partial breast irradiation with brachytherapy using the MammoSite catheter at this institution was WBRT. Partial breast radiotherapy using EBRT was not offered as an alternative at this institution at this time.

### Dosimetric analysis

Dose to the heart was evaluated for all patients with left breast tumors who had a CT scan encompassing the entire heart. Dose to the ipsilateral lung was evaluated for all patients for whom the lung was scanned from apex to diaphragm. DVH analysis was performed and the following parameters were assessed for the MammoSite plan and for the EBRT plan. The maximum (Dmax) and mean (Dmean) doses for each structure were measured. For the heart the highest dose received by 20 cc and 30 cc of the whole heart volume (the D20 and D30 respectively) was measured The volume of the heart receiving a dose of 5 Gy and higher, 10 Gy and higher and 20 Gy and higher (V5, V10 and V20 respectively) were calculated as an absolute volume and as a percentage of the total cardiac volume. The highest dose received by 5 cc (D5) of the ipsilateral lung was measured. The volume of the ipsilateral lung receiving a dose of 10 Gy, 20 Gy and 30 Gy or higher (V10, V20, V30 respectively) was calculated as an absolute volume and as a percentage of the total lung volume. These dosimetric parameters were chosen because they have been shown to correlate with late toxicity in patients undergoing radiotherapy for non small cell lung cancer (NSCLC) [21-24]. The cardiac parameters were chosen to reflect available data regarding the risk of cardiac toxicity following radiation exposure in atomic bomb survivors and following radiotherapy for peptic ulcer disease [25-27].

By convention in breast dosimetry studies the standard nomenclature used is V10 to define the volume receiving 10 Gy and D10 to define the dose received by 10 cc of organ volume. This differs from the standard nomenclature for brachytherapy dosimetry in other areas of the body where the V10 would refer to the volume of tissue receiving 10% or greater of the dose. If comparing the data within this paper to published literature in other primary disease sites, these nomenclature changes should be borne in mind.

### Radiobiologic estimations

It could be considered that use of the standard breast radiotherapy dosimetry reporting parameters for the MammoSite balloon may not be radiobiologically comparable to EBRT since the MammoSite balloon employs a larger dose per fraction than the EBRT. To account for the effect of an increased dose per fraction, the radiobiological equivalents of the standard breast dose reporting parameters were calculated using the linear quadratic equation [28]. Using an alpha/beta (α/β) ratio of 3.6 Gy for breast tumor tissue [29] and 3 Gy for late effects to the heart and lung [30], it was calculated that a MammoSite V4.5 may be equivalent to an EBRT V5, a MammoSite V9 may be equivalent to an EBRT V10, a MammoSite V16.5 may be equivalent to an EBRT V20 and a MammoSite V23.5 may be equivalent to an EBRT V30 (see appendix 1 for biologically equivalent dose (BED) equations). This correction does not account for the dose inhomogeneity produced by a brachytherapy source. However, these effects are likely to be most marked in close proximity to the source and decrease with increasing distance from the source.

The radiobiological effect of an accelerated treatment course was not included in the calculation as it is generally felt that treatment time does not make a radiobiological difference in breast tumors [29]. These calculations also do not use a dose reduction for HDR because these dose reductions may not be as accurate as distance from the catheter increases. If the HDR dose reduction is not used, the most conservative estimate of dose equivalence is obtained since with the HDR dose reduction the dosimetric parameters would be closer to the EBRT measurements than the radiobiologically adjusted dosimetric parameters.

### Statistical Analysis

The dosimetric parameters outlined above were summed and the median calculated. The significance of the difference between the groups was assessed using paired two-tailed Student's *t-*test. The paired *t*-test was chosen because there is a one-to-one pairing between the patient with the MammoSite plan and the EBRT plan because the same CT data sets were used for both analyses.

## Results

Five patients had complete ipsilateral lung CT data and six patients undergoing left breast treatment had complete heart CT data. The median ipsilateral lung volume was 985 cc (range 925–1380 cc). The median cardiac volume was 428 cc (range 337–551 cc).

When comparing the dose received by the ipsilateral lung using the MammoSite catheter and WBRT, the volume of lung irradiated to 20 Gy or more and 30 Gy or more was significantly lower using the MammoSite catheter than WBRT, see table 1. This difference was maintained when the radiobiological effects of an increased dose per fraction were calculated for the V23.5/V30 parameter but only when the volume was considered as a percentage of the whole lung volume for the V16/V20 parameter, see table 2. There was no statistically significant difference in the maximum or mean dose delivered to the lung, though the highest dose received by 5 cc of lung was significantly lower using the MammoSite catheter. There was no statistically significant difference in the volume of lung irradiated to low doses (10 Gy and greater).

**Table 1:** A comparison of doses to the heart and ipsilateral lung dosimetry from the MammoSite catheter and external beam radiotherapy using the standard dosimetric parameters.

Dosimetric Parameters	MammoSite (34 Gy/10#/1 week)		External Beam (50 Gy/25#/5 weeks)		p-value
	Mean	Range	Mean	Range	

**Lung **n = 5					
Dmean	4.2 Gy	2.1–5.6 Gy	6.5 Gy	2.6–11.8 Gy	p = 0.18
Dmax	31 Gy	6.3–45.7 Gy	51.6 Gy	49.7–55.9 Gy	p = 0.06
V30 (cc)	2 cc	0–7.2 cc	86.7 cc	31.5–184 cc	p = 0.04*
V30 (%)	0.2%	0–0.5%	8.3%	2.3–19.9%	p = 0.04*
V20 (cc)	13.4 cc	0–34.6 cc	105 cc	46.1–205 cc	p = 0.04*
V20 (%)	1.3%	0–2.5%	10%	3.3–22.2%	p = 0.04*
V10 (cc)	83.3 cc	0–170 cc	133.6 cc	67.8–235 cc	p = 0.31
V10 (%)	8%	0–13.3%	12.8%	4.9–25.4%	p = 0.20
D5	11.3 Gy	3.9–15.3 Gy	49.9 Gy	47.5–53.4 Gy	p = 0.005*

**Heart**					
Dmean	3.8 Gy	3.1–4.8 Gy	3.5 Gy	1.1 – 5.1 Gy	p = 0.63
Dmax	16.6 Gy	10.6–27.2 Gy	44.1 Gy	15.5–51.8 Gy	p = 0.004*
V20 (cc)	0.3 cc	0–1.5 cc	15.6 cc	0–23.9 cc	p = 0.008*
V20 (%)	0.1%	0–0.3%	3.7%	0–5.3%	p = 0.001*
V10 (cc)	10.6 cc	0.2–33.6 cc	22.9 cc	0.3–39.0 cc	p = 0.07
V10 (%)	2.5%	0–7.5%	5.4%	0.1–8.7%	p = 0.07
V5 (cc)	83.8 cc	46.9–145.0 cc	41.5 cc	3.1–82.3 cc	p = 0.006*
V5 (%)	19.8%	11.0–32.2%	9.6%	0.8–18.3%	p = 0.008*
D20	8.5 Gy	6.4–11.9 Gy	15.3 Gy	2.3–24.3 Gy	p = 0.10
D30	7.5 Gy	5.8–10.4 Gy	7.6 Gy	2–14.0 Gy	p = 0.94

* represents a statistically significant p-value

**Table 2:** A comparison of doses to the heart and ipsilateral lung dosimetry from the MammoSite catheter and external beam radiotherapy using the radiobiologically adjusted dosimetric parameters.

MammoSite (34 Gy/10#/1 week)		External Beam (50 Gy/25#/5 weeks)		p-value
Mean	Range	Mean	Range	

Lung				
**V23.5 **7 cc	0–20.4 cc	**V30 **86.7 cc	31.5–184 cc	p = 0.05*
**V23.5 **0.7%	0–1.5%	**V30 **8.3%	2.3–19.9%	p = 0.03*
**V16.5 **23.6 cc	0–57.9 cc	**V20 **105 cc	46.1–205 cc	p = 0.06
**V16.5 **2.3%	0–4.2%	**V20 **10%	3.3–22.2%	p = 0.03*
**V9 **98.1 cc	0–201.7 cc	**V10 **133.6 cc	67.8–235 cc	p = 0.48
**V9 **9.4%	0–13.3%	**V10 **12.8%	4.9–25.4%	p = 0.26

Heart				
**V16.5 **1.2 cc	0–5.2 cc	**V20 **15.7 cc	0–23.9 cc	p = 0.009*
**V16.5 **0.3%	0–1.2%	**V20 **3.7%	0–5.3%	p = 0.01*
**V9 **15.9 cc	2.1–47.4 cc	**V10 **22.9 cc	0.3–39.0 cc	p = 0.34
**V9 **3.7%	0.5–10.5%	**V10 **5.4%	0.1–8.7%	p = 0.35
**V4.5 **105.7 cc	64.5–173.5 cc	**V5 **41.47 cc	3.1–82.3 cc	p = 0.001*
**V4.5 **25.0%	15.1–38.5%	**V5 **9.6%	0.8–18.3%	p = 0.002*

* represents a statistically significant p-value

When comparing the dose received by the heart using the MammoSite catheter and WBRT, the MammoSite catheter delivered a significantly lower maximum dose than WBRT, see table 1. The volume of the heart irradiated to 20 Gy or more was significantly higher using WBRT than the MammoSite catheter, see table 1. This statistically significant difference was maintained when the radiobiological effects of an increased dose per fraction were calculated, see table 2. There was no statistically significant difference in the volume of the heart irradiated to 10 Gy and greater and no difference in the mean cardiac dose. When much lower doses to the heart were examined (V5) it could be seen that the WBRT delivered significantly lower radiation than the MammoSite catheter, see table 1. This significant difference was maintained when radiobiological adjustment was undertaken, see table 2.

## Discussion

This study gives low values of radiation dose using the MammoSite catheter for both heart and lung. In all cases less than 25% of the ipsilateral lung received less than 20 Gy, which has been shown to correlate with a lower incidence of late toxicity in lung cancer patients [21-24]. The maximum dose received by the heart and the maximum dose received by 5 cc of lung were significantly lower with the MammoSite technique than EBRT. The MammoSite catheter irradiated a significantly lower volume of the heart and lung to higher doses than EBRT. The volume of lung irradiated to lower doses was similar with both techniques. However, the volume of heart irradiated to lower doses was significantly higher with the MammoSite catheter than WBRT. When radiobiological adjustment was performed, these significant differences were maintained.

The planning target volume (PTV) irradiated is much lower with the MammoSite catheter than EBRT and it could be argued that the MammoSite technique would be expected to deliver a lower dose of radiation to critical normal tissues. However, it is interesting that the volumes of heart and lung irradiated to moderately low doses are not significantly different, possibly due to the radial dose distribution using single catheter brachytherapy. It is also interesting that the volume of heart irradiated to very low doses is higher with the MammoSite technique than WBRT. In certain clinical situations, the MammoSite catheter may deliver higher doses of radiation to clinically significant levels than WBRT, such as a left-sided implant lying directly on the chest wall over the heart. In these situations it may be preferable to use multi-catheter brachytherapy implants which give the ability to sculpt the dose around organs at risk. The ability to identify these patients prior to implant placement would be valuable.

Early studies of breast cancer radiotherapy showed an increased cardiac mortality in patients undergoing irradiation of the left breast [31,32]. In Hodgkin's disease mediastinal irradiation exceeding 30 Gy (V30) is associated with an increased risk of death from cardiac disease, however in the present study no patient undergoing MammoSite brachytherapy had any cardiac volume receiving over 30 Gy; in fact only 1 patient had irradiation to the heart exceeding 20 Gy. Due to the long latency of cardiac morbidity following radiotherapy for breast cancer and the relatively new advent of image-guided radiotherapy planning, no distinct CT-based dosimetric parameters for the heart have been correlated with late effect following breast cancer radiotherapy. Although the risk of late cardiovascular events and irradiation to higher doses has long been associated with EBRT, more recently there has been evidence that there is an increased risk of radiation-induced heart disease at lower levels of exposure [25,27]. Also the risk of radiation induced tumors following exposure to low doses of radiation must always be remembered [33].

The percentage of the left ventricle (LV) irradiated correlates with the development of perfusion defects on cardiac SPECT (single photon emitting computed tomography) scanning [34]. But it is unknown whether a potentially reversible cardiac perfusion defect is associated with later myocardial morbidity. An elevated body mass index (BMI) is also associated with increased LV perfusion defects, possibly because these patients have significantly higher rates of radiotherapy set-up errors resulting in increased LV irradiation [34]. Brachytherapy eliminates set-up errors due to the delivery of dose within catheters fixed in the tissue, which may make it a preferred option in women with early breast cancer and a high BMI.

In patients with lung cancer, it has been shown that late toxicity rates increase as the volume of lung receiving over 20 Gy increases [21,22]. When this parameter was assessed by Lind *et al. *in patients undergoing whole breast EBRT for breast cancer, the same association was seen [35]. Therefore it is recommended that the pulmonary V20 be kept below 30%. Increasing age and a pre-existing decrease in pulmonary capacity was also shown to influence late pulmonary toxicity [35]. This emphasizes the importance of considering all associated co-morbidities when assessing the risk of late toxicities rather than just isolated dosimetric parameters.

When the dosimetry of the MammoSite catheter has been compared to other EBRT techniques, both partial and whole breast, the majority of studies have shown lower volumes of heart and lung receiving high doses of radiation with the MammoSite catheter [19,36,37]. Patients who had a higher cardiac dose using the MammoSite catheter than a whole breast IMRT technique appeared to be those whose tumor bed lay close to the chest wall [36]. However, a definitive "safe" distance from the chest wall could not be determined. In the current study, it could be subjectively assessed that in women with large breasts, with the catheter lying far from the chest wall, the cardiac doses were minimal or undetectable. The cardiac dose is dependent on factors which can be modified by a change in the radiation technique such as the position of the catheter within the breast, proximity to the chest wall or medial versus lateral placement and also factors which cannot be modified such as the position of the heart within the thorax which can vary greatly from patient to patient.

Khan *et al. *showed that the MammoSite catheter resulted in significantly higher cardiac V_5 _(5% or greater of prescription dose) due to the ability to shape the dose using EBRT techniques. Although the current study showed much lower values for cardiac V10 and V20 (and their radiobiologically adjusted equivalents) using the MammoSite catheter than would be extrapolated from Khan *et al.*'s results, the decrease in irradiation to lower doses may still have marked clinical significance [25-27]. The conformation of the MammoSite catheter with dose delivery via a single catheter means that there is no opportunity for "dose sculpting" (conforming the dose to the PTV) to decrease the dose received by the heart in situations where the balloon lies closer to the chest wall without compromising PTV coverage. This problem may be overcome in the future with the introduction of single entry insertion catheters with multiple channels such as the ClearPath™ catheter [38].

Limitations of this study include the use of the CT treatment planning images with the MammoSite balloon inflated for the EBRT dosimetry which may result in a larger breast volume than if the EBRT was planned without the MammoSite catheter in situ. However, this increased volume is more likely to affect hot spots within the breast than heart and lung doses since these are mainly affected by curvature of the chest wall and the position of the tumor bed within the breast. In contrast, in studies where the MammoSite catheter is simulated within a seroma cavity on an EBRT CT planning scan, treatment volumes and thus normal tissue dosimetry may be underestimated because the MammoSite balloon causes tissue displacement and stretching resulting in a larger PTV than the PTV encompassed by 1 cm around the seroma [39,40]. The effect of this displacement could be difficult to predict, even with the use of a pre-MammoSite insertion CT scan. The CT images were obtained without the use of an angled breast board, which could result in higher doses delivered using the EBRT plans than if it modeled using standard treatment techniques. However, this was compensated for within the EBRT planning process with the use of collimator angulation. Cardiac blocks were not placed for the EBRT plans. Use of these may have resulted in slightly lower cardiac doses, as could other techniques such as active breathing control.

The dosimetric parameters that have been assessed are based on large datasets of external beam radiotherapy patients who would have received treatment using conventional fractionation schemes of 1.8–2 Gy per fraction. Where radiobiological correction has been made, it must be remembered that the BED equation does not fully account for the effects of a higher dose per fraction for the critical normal structures. The effect on the heart and lungs of a higher dose per fraction are unknown, especially in the clinical scenario of a patient proceeding to potentially cardiotoxic chemotherapy.

## Conclusion

This small subset study gives low values for cardiac and lung normal tissue doses using the MammoSite applicator using both standard measuring parameters and biologically equivalent parameters. When compared to whole breast EBRT fields, the volume of the heart and lung receiving higher doses of radiation is significantly lower using the MammoSite catheter. The volume of heart receiving low doses of radiation is significantly higher using the MammoSite catheter. It is unknown which radiation exposure may be the most clinically significant, higher doses or lower doses. The volume of lung receiving low doses of radiation is similar with both techniques. Long-term prospective follow-up would be of value in this group of patients to correlate dose received with late toxicity paying attention to the late effects of an increased dose per fraction using this technique. Ongoing research will focus on OAR dosimetry using EBRT and IMRT techniques versus MammoSite and other brachytherapy techniques, with particular focus on the effect of differing position of the MammoSite catheter within the breast.

## Competing interests

Dr Stewart received a resident travel grant from Nucletron and Cytec.

All other authors declare no conflicts of interest.

## Authors' contributions

AS was involved in conception and design, acquisition and analysis of data, manuscript writing. DOF, JH, RC were involved in design, acquisition and analysis of data. AK, SM, PD were involved in conception, design and manuscript writing. All authors have read and approved the final manuscript.

## Appendix 1

### Biologic equivalent dose (BED) calculations

BED = *d *× *n *[1 + (*d*/α/β)]

Where *d *= dose per fraction

*n *= total number of fractions delivered

*nd *= D where D = total dose

If the α/β for late toxicity to the heart and lungs is taken to be 3 Gy [30] the following equations were derived to calculate an equivalent probability of late effects:

For a V10 with a total dose of 50 Gy/25 fractions, the dose per fraction is 0.4 Gy

Thus EBRT BED = 10 × (1 + 0.4/3)

= 11.3

MS BED = 9 × (1 + 0.9/3)

= 11.7 Gy_3_

Hence a V9 using 34 Gy/10 fractions is equivalent to a V10 using 50 Gy/25 fractions (the equivalent dosimetry points were calculated to the nearest 0.5 Gy for ease of measurement).

V5 EBRT BED = 5 × (1 + 0.2/3)

= 5.3 Gy_3_

V4.5 equivalent MS BED = 4.5 × (1 + 0.45/3)

= 5.2 Gy_3_

V20 EBRT BED = 20 × (1 + 0.8/3)

= 25.3 Gy_3_

V16.5 equivalent MS BED = 16.5 × (1 + 1.65/3)

= 25.6 Gy_3_

V30 EBRT BED = 30 × (1 + 1.2/3)

= 42 Gy_3_

V23.5 equivalent MS BED = 23.5 × (1 + 2.35/3)

= 41.9 Gy_3_

## References

[B1] Sanders ME, Scroggins T, Ampil FL, Li BD (2007). Accelerated partial breast irradiation in early-stage breast cancer. J Clin Oncol.

[B2] Vicini FA, Beitsch PD, Quiet CA, Keleher A, Garcia D, Snider HC, Gittleman MA, Zannis VJ, Kuerer H, Whitacre EB, Whitworth PW, Fine RE, Haffty BG, Stolier A, Arrambide LS (2005). First analysis of patient demographics, technical reproducibility, cosmesis, and early toxicity: results of the American Society of Breast Surgeons MammoSite breast brachytherapy trial. Cancer.

[B3] Arthur DW, Koo D, Zwicker RD, Tong S, Bear HD, Kaplan BJ, Kavanagh BD, Warwicke LA, Holdford D, Amir C, Archer KJ, Schmidt-Ullrich RK (2003). Partial breast brachytherapy after lumpectomy: Low-dose-rate and high-dose-rate experience. Int J Radiat Oncol Biol Phys.

[B4] Baglan KL, Martinez AA, Frazier RC, Kini VR, Kestin LL, Chen PY, Edmundson G, Mele E, Jaffray D, Vicini FA (2001). The use of high-dose-rate brachytherapy alone after lumpectomy in patients with early-stage breast cancer treated with breast-conserving surgery. Int J Radiat Oncol Biol Phys.

[B5] Benitez PR, Streeter O, Vicini F, Mehta V, Quiet C, Kuske R, Hayes MK, Arthur D, Kuerer H, Freedman G, Keisch M, Dipetrillo T, Khan D, Hudes R (2006). Preliminary results and evaluation of MammoSite balloon brachytherapy for partial breast irradiation for pure ductal carcinoma in situ: a phase II clinical study. Am J Surg.

[B6] Chen PY, Vicini FA, Benitez P, Kestin LL, Wallace M, Mitchell C, Pettinga J, Martinez AA (2006). Long-term cosmetic results and toxicity after accelerated partial-breast irradiation: a method of radiation delivery by interstitial brachytherapy for the treatment of early-stage breast carcinoma. Cancer.

[B7] Dickler A, Kirk MC, Chu J, Nguyen C (2005). The MammoSite breast brachytherapy applicator: A review of technique and outcomes. Brachytherapy.

[B8] Edmundson GK, Vicini FA, Chen PY, Mitchell C, Martinez AA (2002). Dosimetric characteristics of the MammoSite RTS, a new breast brachytherapy applicator. Int J Radiat Oncol Biol Phys.

[B9] Harper JL, Jenrette JM, Vanek KN, Aguero EG, Gillanders WE (2005). Acute complications of MammoSite brachytherapy: a single institution's initial clinical experience. Int J Radiat Oncol Biol Phys.

[B10] Keisch M, Vicini F, Kuske RR, Hebert M, White J, Quiet C, Arthur D, Scroggins T, Streeter O (2003). Initial clinical experience with the MammoSite breast brachytherapy applicator in women with early-stage breast cancer treated with breast-conserving therapy. Int J Radiat Oncol Biol Phys.

[B11] Keisch M, Vicini F, Scroggins T, Hebert M, White J, Kuske R, Quiet C, Arthur D, Streeter O (2005). Thirty-nine month results with the MammoSite brachytherapy applicator: Details regarding cosmesis, toxicity and local control in partial breast irradiation. Int J Radiat Oncol Biol Phys.

[B12] Polgár C, Sulyok Z, Fodor J, Orosz Z, Major T, Takácsi-Nagy Z, Mangel LC, Somogyi A, Kásler M, Németh G (2002). Sole brachytherapy of the tumor bed after conservative surgery for T1 breast cancer: Five-year results of a phase I/II study and initial findings of a randomized phase III trial. J Surg Oncol.

[B13] Shah NM, Tenenholz T, Arthur D, DiPetrillo T, Bornstein B, Cardarelli G, Zheng Z, Rivard MJ, Kaufman S, Wazer DE (2004). MammoSite and interstitial brachytherapy for accelerated partial breast irradiation. Cancer.

[B14] Zannis V, Beitsch P, Vicini F, Quiet C, Keleher A, Garcia D, Snider H, Gittleman M, Kuerer H, Whitacre E, Whitworth P, Fine R, Haffty B, Stolier A, Mabie J (2005). Descriptions and Outcomes of insertion techniques of a brachytherapy balloon catheter in 1403 patients enrolled in the American Society of Breast Surgeons MammoSite breast brachytherapy registry trial. Am J Surg.

[B15] King TA, Bolton JS, Kuske RR, Fuhrman GM, Scroggins TG, Jiang XZ (2000). Long-term results of wide-field brachytherapy as the sole method of radiation therapy after segmental mastectomy for T(is,1,2) breast cancer. Am J Surg.

[B16] Chao KK, Vicini FA, Wallace M, Mitchell C, Chen P, Ghilezan M, Gilbert S, Kunzman J, Benitez P, Martinez A (2007). Analysis of treatment efficacy, cosmesis, and toxicity using the MammoSite breast brachytherapy catheter to deliver accelerated partial breast irradiation: The William Beaumont Hospital experience. Int J Radiat Oncol Biol Phys.

[B17] Vicini FA, Kestin L, Chen P, Benitez P, Goldstein NS, Martinez A (2003). Limited-field radiation therapy in the management of early-stage breast cancer. J Natl Cancer Inst.

[B18] Khan AJ, Kirk MC, Mehta PS, Seif NS, Griem KL, Bernard DA, Chu JCH, Dickler A (2006). A dosimetric comparison of three-dimensional conformal, intensity-modulated radiation therapy and MammoSite partial-breast irradiation. Brachytherapy.

[B19] Garza R, Albuquerque K, Sethi A (2006). Lung and cardiac tissue doses in left breast cancer patients treated with single-source breast brachytherapy compared to external beam tangent fields. Brachytherapy.

[B20] Astrahan MA, Jozsef G, Streeter OE (2004). Optimization of MammoSite therapy. Int J Radiat Oncol Biol Phys.

[B21] Yorke ED, Jackson A, Rosenzweig KE, Merrick SA, Gabrys D, Venkatraman ES, Burman CM, Leibel SA, Ling CC (2002). Dose-volume factors contributing to the incidence of radiation pneumonitis in non-small-cell lung cancer patients treated with three-dimensional conformal radiation therapy. Int J Radiat Oncol Biol Phys.

[B22] Graham MV, Purdy JA, Emami B, Harms W, Bosch W, Lockett MA, Perez CA (1999). Clinical dose-volume histogram analysis for pneumonitis after 3D treatment for non small cell lung cancer. Int J Radiat Oncol Biol Phys.

[B23] Hernando ML, Marks LB, Bentel GC, Zhou SM, Hollis D, Das SK, Fan M, Munley MT, Shafman TD, Anscher MS, Lind PA (2001). Radiation-induced pulmonary toxicity: A dose-volume histogram analysis in 201 patients with lung cancer. Int J Radiat Oncol Biol Phys.

[B24] Marks LB, Kocak Z, Zhou S, Yu X, Light K, Anscher MS, Kahn D, Wong T, Folz RJ, Hollis D (2005). The association between the mean heart dose, mean lung dose, tumor location and RT associated heart and lung toxicity. Int J Radiat Oncol Biol Phys.

[B25] McGale P, Darby SC (2005). Low doses of ionising radiation and circulatory diseases: a systematic review of the published epidemiological evidence. Radiat Res.

[B26] Preston DL, Shimizu Y, Pierce DA, Suyama A, Mabuchi K (2003). Studies of mortality of atomic bomb survivors. Report 13: solid cancer and noncancer disease mortality: 1950–1997. Radiat Res.

[B27] Carr ZA, Land CE, Kleinerman RA, Weinstock RW, Stovall M, Griem ML, Mabuchi K (2005). Coronary heart disease after radiotherapy for peptic ulcer disease. Int J Radiat Oncol Biol Phys.

[B28] Joiner MC, Kogel AJ van der, Steel GG (1997). The linear quadratic approach to fractionation and calculation of isoeffect relationships. Basic Clinical Radiobiology.

[B29] Tutt A, Yarnold J (2006). Radiobiology of Breast Cancer. Clin Onc.

[B30] Kogel AJ van der, Steel G (1997). Radiation response and tolerance of normal tissues. Basic Clinical Radiobiology.

[B31] Early Breast Cancer Trialists' Collaborative Group (2000). Favourable and unfavourable effects on long-term survival of radiotherapy for early breast cancer. Lancet.

[B32] Cuzick H, Stewart L, Rutqvist J, Houghton J, Edwards R, Redmond C, Peto R, Baum M, Fisher B, Host H (1994). Cause-specific mortality in long-term survivors of breast cancer who participated in trials of radiotherapy. J Clin Oncol.

[B33] Hall EJ, Wuu CS (2003). Radiation-induced second cancers: the impact of 3D-CRT and IMRT. Int J Radiat Oncol Biol Phys.

[B34] Evans ES, Prosnitz RG, Yu X, Zhou SM, Hollis DR, Wong TZ, Light KL, Hardenbergh PH, Blazing MA, Marks LB (2006). Impact of patient-specific factors, irradiated left ventricular volume and treatment set-up errors on the development of myocardial perfusion defects after radiation therapy for left-sided breast cancer. Int J Radiat Oncol Biol Phys.

[B35] Lind PARM, Wennberg B, Gagliardi G, Fornander T (2001). Pulmonary complications following different radiotherapy techniques for breast cancer and the association to irradiated lung volume and dose. Breast Cancer Res Treat.

[B36] Tsai AF, McNeeley S, Li T, Anderson P, Freedman G (2006). A comparison of heart doses in MammoSite brachytherapy and breast IMRT. Int J Radiat Oncol Biol Phys.

[B37] Weed DW, Edmundson GK, Vicini FA, Chen PY, Martinez AA (2005). Accelerated partial breast irradiation: A dosimetric comparison of three different techniques. Brachytherapy.

[B38] ClearPath. http://www.clearpath.net.

[B39] Major T, Niehoff P, Kovacs G, Fodor J, Polgar C (2006). Dosimetric comparisons between high dose rate interstitial and MammoSite balloon brachytherapy for breast cancer. Radiother Oncol.

[B40] Dickler A, Kirk M, Choo J, Hsi WC, Chu J, Dowlatshahi K, Francescatti D, Nguyen C (2004). Treatment volume and dose optimization of MammoSite breast brachytherapy applicator. Int J Radiat Oncol Biol Phys.

